# Examining the Progressive Behavior and Neuropathological Outcomes Associated with Chronic Repetitive Mild Traumatic Brain Injury in Rats

**DOI:** 10.1093/texcom/tgaa002

**Published:** 2020-02-20

**Authors:** Eric Eyolfson, Glenn R Yamakawa, Yannick Griep, Reid Collins, Thomas Carr, Melinda Wang, Alexander W Lohman, Richelle Mychasiuk

**Affiliations:** 1 Department of Psychology, Alberta Children’s Hospital Research Institute, The University of Calgary, Calgary, AB, T2N 1N4, Canada; 2 Department of Psychology, Hotchkiss Brain Institute, The University of Calgary, Calgary, AB, T2N 1N4, Canada; 3 Department of Neuroscience, Central Clinical School, Monash University, Melbourne, VIC, 3004, Australia; 4 Division of Epidemiology, Stress Research Institute, Stockholm University, 106 91 Stockholm, Sweden; 5 Behavioral Science Institute, Radbound University, 9104, 6500 HE, Nijmegen, The Netherlands; 6 Department of Cell Biology and Anatomy, Alberta Children’s Hospital Research Institute, The University of Calgary, Calgary, AB, T2N 1N4, Canada; 7 Department of Cell Biology and Anatomy, Hotchkiss Brain Institute, The University of Calgary, Calgary, AB, T2N 1N4, Canada

**Keywords:** adolescence, concussion, GFAP, IBA1, sex differences, telomere length

## Abstract

While the physical and behavioral symptomologies associated with a single mild traumatic brain injury (mTBI) are typically transient, repetitive mTBIs (RmTBI) have been associated with persisting neurological deficits. Therefore, this study examined the progressive changes in behavior and the neuropathological outcomes associated with chronic RmTBI through adolescence and adulthood in male and female Sprague Dawley rats. Rats experienced 2 mTBIs/week for 15 weeks and were periodically tested for changes in motor behavior, cognitive function, emotional disturbances, and aggression. Brain tissue was examined for neuropathological changes in ventricle size and presentation of Iba1 and GFAP. We did not see progressively worse behavioral impairments with the accumulation of injuries or time, but did find evidence for neurological and functional change (motor disturbance, reduced exploration, reduced aggression, alteration in depressive-like behavior, deficits in short-term working memory). Neuropathological assessment of RmTBI animals identified an increase in ventricle size, prolonged changes in GFAP, and sex differences in Iba1, in the corpus callosum, thalamus, and medial prefrontal cortex. Telomere length reduced exponentially as the injury load increased. Overall, chronic RmTBI did not result in accumulating behavioral impairment, and there is a need to further investigate progressive behavioral changes associated with repeated injuries in adolescence and young adulthood.

## Introduction

Traumatic brain injury (TBI) is one of the most under-addressed and expensive health problems in North America (Prins et al. 2010). Mild TBIs (mTBI) account for the majority of brain injuries (Yeates 2010; Prins et al. 2013) and incur significant global costs each year (Smith 2006). Although considered mild, these injuries initiate a neurophysiological cascade that results in impaired vascular, inflammatory, metabolic, and neuronal function (Hicks et al. 1993; Gao and Chen 2011; Gosselin et al. 2011; Redell et al. 2013; Fehily and Fitzgerald 2017; Kim et al. 2017; Wendel et al. 2018). While the physical and behavioral symptomologies associated with a single mTBI are typically transient, repetitive mTBIs (RmTBI) have been associated with persisting neurological deficits and neurodegenerative diseases (Uryu et al. 2002; Huh et al. 2007; Baugh et al. 2012; Smith et al. 2013). Moreover, a history of mTBI increases the risk of a subsequent mTBI, leading to exacerbated symptom presentation and a protracted trajectory for recovery (i.e., post-concussion syndrome) (Silverberg et al. 2013; Iverson et al. 2017). Recent clinical evidence also indicates that the potential for cumulative effects is greatest when mTBIs occur in short succession (Silverberg et al. 2013). Therefore, individuals at high risk for experiencing RmTBI are of particular concern.

Medical interest in the long-term effects of RmTBI is not new and began at least a century ago with the description of punch drunk syndrome in boxers (Martland 1928; Critchley 1949). Descriptions of traumatic encephalopathy and dementia pugilistica linked to cumulative and chronic TBI in boxers were often associated with neurological deficits while the boxer was still alive (Johnson 1969). The term chronic traumatic encephalopathy (CTE) is used to describe this phenomenon and has been reported in individuals who participate in high-collision sports such as football, hockey, rugby, soccer, and wrestling, as well as in the cases of domestic violence and among military personnel (Asken et al. 2017). Although highly disputed, the behavioral symptomologies associated with clinical presentation of CTE include depression, anxiety, suicidality, cognitive impairment, anger problems, sleep disturbances, headache, body pain, and marital problems (McKee et al. 2009; Gavett et al. 2011; Omalu et al. 2011; Baugh et al. 2012; McKee et al. 2013). While the neuropathological characteristics attributed to CTE are more agreed upon than the clinical presentation, they are still often contentious and diverse and include cortical atrophy, axonal varicosity, ventricle enlargement, cavum septum pellucidum, and accumulation of phosphorylated-tau (p-tau) (McKee et al. 2009, 2016; Baugh et al. 2012).

Despite the fact that the clinical syndrome of CTE has not yet been clearly identified, its prevalence is unknown, and the current neuropathological parameters are preliminary at best, there is mounting public concern across the world that even a single mTBI may lead to neurodegenerative disorders later in life. Although some evidence links RmTBI to the development of neurodegenerative conditions, these findings have been highly contested, especially in the context of CTE (Randolph 2018; Smith et al. 2019; Stewart et al. 2019). Given that it is challenging to study the development and progression of CTE in the clinical setting because it is only diagnosable upon autopsy, and there is no understanding of the extent and distribution of CTE pathology required to produce neurological dysfunction or to distinguish diseased from healthy tissue (Iverson et al. 2018), this study sought to use a preclinical rodent model to examine the effects of chronic RmTBI on behavioral and neuropathological outcomes. As it is difficult to model tau pathologies in rodents that have not been genetically altered to express human tau, we sought to examine allograph inflammatory factor 1 (Iba1) and glial fibrillary acidic protein (GFAP) neuropathology. These 2 important glial-specific proteins have been used extensively to track and quantify astrogliosis.

Therefore, the purpose of this study was to examine the progressive changes in behavior, and the neuropathological outcomes, associated with chronic RmTBI through adolescence and early adulthood in male and female Sprague Dawley rats. In an effort to mimic ethological conditions associated with high-collision sports, male and female Sprague Dawley rats experienced 2 mTBIs per week for 15 weeks and were periodically tested for changes in motor behavior, cognitive function, emotional disturbances (anxiety- and depressive-like behaviors), and aggression. At completion of the study, we used immunohistological techniques to examine brain tissue for neuropathological changes in ventricle size and presentation of Iba1 and GFAP.

## Materials and Methods

### Animals and RmTBI Procedures

The experiments and procedures described below were approved by the University of Calgary Conjoint Facilities Research Ethics Board and were conducted in accordance with the Canadian Council of Animal Care. Twenty-four Sprague Dawley rats (16 male, 8 female) were obtained from Charles River Laboratories and were housed in same-sex groups of 4. All rats had *ad libitum* access to food and water and were maintained on a 12:12 h light/dark cycle (lights on at 0700) in a temperature-controlled (21 °C) husbandry room. RmTBIs were administered twice a week (spaced 3 or 4 days apart) for 15 weeks, beginning at postnatal day 34 (P34) and concluding on P133. Each rat in the RmTBI group (10 male, 5 female) received a total of 30 mTBIs. Each of the mTBIs was administered with the lateral impact device, as previously described by our research group (Mychasiuk et al. 2016a, 2016b). In brief, rats were lightly anesthetized (~30 s) with isoflurane gas and placed in the prone position on a Teflon board. A 50-g weight was propelled towards the rats’ head using pneumatic air pressure. When the weight impacted the aluminum helmet, the rat was propelled into a 180° horizontal rotation. The aluminum helmet is designed to prevent skull damage while still ensuring rotational, acceleration, and deceleration forces are imposed on the brain. The impact speed for the first 10 mTBIs was 7.9 ± 0.17 m/s (~63 G) and was increased to 9.01 ± 0.15 m/s (~82.75 G) for the final 20 mTBIs. The impact speed was increased following the 10th mTBI (at ~P100) as rats had grown substantially, and this would represent the increased impact speeds one would expect to see as humans transition from adolescents and adults. Rats in the sham group (6 male, 3 female) were lightly anesthetized each time, placed in prone position on the Teflon board, but did not receive the impact. Following each mTBI or sham procedure, the *time-to-right* (the time each rat took to right itself from a supine to prone position), was recorded and used as a measure of loss of consciousness.

### Behavioral Testing

Behavioral testing was carried out at various time intervals throughout the study to examine progressive changes in balance and motor coordination, cognitive function, and emotional processing as a function of increasing mTBIs.

#### Beam Walking

Animals were tested in a beam walking task similar to that described by Schallert et al. (2002) at four distinct time points: P50, P85, P109, and P134 (following the 5th, 15th, 22nd, and 30th mTBI, respectively). The rat’s home cage was placed at one end of a 165-cm-long tapered beam. The initial trial on each day was used as a learning trial whereby the rat had to learn to walk from the start to the home cage. The rat was given 4 additional videotaped trials, where a researcher blinded to experimental conditions scored the time it took the rat to cross the beam.

#### Open Field

General locomotor activity for each rat was tested in the open field at 4 distinct time points: P51, P86, P113, and P135 (following the 5th, 15th, 23rd, and 30th mTBI, respectively). Rats were individually placed into a circular arena (134 cm diameter) and allowed to explore their environment for 10 min. An overhead tracking camera and Noldus EthoVision XT10 software was used to track the rat’s movement and measure distance traveled.

#### Elevated Plus Maze

The elevated plus maze (EPM) was used to examine anxiety-like behavior at 4 time points: P56, P92, P114, and P136 (following the 7th, 17th, 23rd, and 30th mTBI, respectively). The EPM was black Plexiglas, elevated 55 cm above the ground and consisted of 2 open arms and 2 closed arms. Rats were placed in the center of the EPM and videotaped for 5 min. A research associate blinded to the experimental conditions scored the amount of time each rat spent in the open and closed arms.

#### Novel Context Mismatch

The novel context mismatch (NCM) was used to assess cognitive function and changes in short-term working memory at 3 time points throughout the study. A protocol similar to that described by Spanswick and Sutherland (2010) was employed. For each NCM testing session, a single probe trial followed 3 training days. The probe trials occurred on P62, P98, and P151 (following the 8th, 18th, and 30th mTBI, respectively). A research associate blinded to the experimental conditions scored the amount of time the rat spent with the old and new/novel object.

#### Forced Swim

Depressive-like behavior was assessed with the forced swim (FS) task using a protocol similar to that described by Yadid et al. (2001), on P102 and P141 (following the 20th and 30th mTBI, respectively). The rat was placed in a cylindrical tank (30 cm diameter × 60 cm deep) filled with warm water (~25 °C). The rat was videotaped for 7 min and a research associate blinded to the experimental conditions scored the amount of time the rat spent immobile.

#### Dominance Tube

Social status/aggressive-like behavior was assessed in the dominance tube (DT) on P152 and P153 (following the 30th mTBI). To measure changes in social status or aggression, same-sex rats were released into opposite ends of a clear tube, narrow enough so that the animals could not turn around. The rats meet in the middle, and the more aggressive animal would display dominance by pushing forward to force their opponent to withdraw/retreat out of the tube. An animal was declared the loser if all 4 of its paws were out of the tube and the winner was the one remaining inside. There was a total of 3 trials per day; on the 1st day, rats faced an animal with a similar injury profile (RmTBI vs. RmTBI); on the 2nd day, the rats faced an animal with the opposite injury profile (RmTBI vs. Sham). Win percentage was recorded for each animal.

### Telomere Length Analysis

An ear notch sample was collected from each rat at P34, P96, and P158 (prior to the 1st injury, following the 17th mTBI, and at euthanasia). Genomic DNA was extracted from the ear notch tissue using Sigma RedExtract N-Amp Tissue PCR Kit according to the manufacturer’s specifications. The quantity and quality of DNA were measured with Nanodrop 2000 (Thermo Fisher Scientific, Waltham, MA, USA). Telomere length analysis was conducted as previously described by our laboratory (Hehar and Mychasiuk 2016).

### Neuropathology

On P158, following completion of all behavioral testing (25 days after the last injury), rats were injected with an overdose of sodium pentobarbital and were intracardially perfused with approximately 200 mL of phosphate-buffered saline (0.1 M PBS) followed by a similar volume of 4% paraformaldehyde (PFA). Brains were extracted and stored in 4% PFA for 24 h after which they were transferred to a 30% sucrose, 0.1 M PBS solution. Upon sinking, brains were sectioned using a freezing sliding microtome. Sections were taken employing a sampling fraction of 1/12 and were cut at a thickness of 40 microns.

#### Cresyl Violet

We used the Cavalieri method to examine changes in size of the lateral and third ventricle. A single series of tissue was stained with cresyl violet using standard laboratory procedures (1% cresyl violet acetate in distilled water), dehydrated, cleared, and cover-slipped with permount. Images of brain sections were captured using a Zeiss Axioplan 2 microscope attached to a Zeiss Axiocam 503 camera with a 5×/0.25 objective. Using ImageJ software (https://imagej.nih.gov/ij/) a grid (grid size = 0.05 mm^2^, 141.5 pixels = 1 mm) was placed across 14–15 brain sections (beginning at the first presentation of the third ventricle) for point counting.

#### Immunohistochemistry and Cell Counting

Free-floating sections were incubated with primary rabbit anti-Iba1 (1:1000; Abcam—019-19 741) or rabbit anti-GFAP (1:1000; Abcam—7260) antibodies in 0.1 M PBS, 0.3% Triton-X for 24 h. Sections were then washed 3× in 0.1 M PBS and incubated with secondary biotinylated goat anti-rabbit (1:1000; Vector, BA—1000) and Alexa Fluor488 goat anti-rabbit (1:500; Abcam—111-545-003), respectively, for 24 h. Tissue was counterstained with 4,6-Diamidino-2-phenylindole (Sigma Aldrich) and then mounted and cover-slipped using a fluorescent mounting medium, and stored at 4 °C until quantification.

Confocal z-stacks from the thalamus, corpus callosum, and ipsilateral/contralateral medial prefrontal cortex were collected on a Nikon A1 laser scanning microscope at ×20 magnification and 1.5 um intervals. Max intensity projections of either Iba1 or GFAP channels were imported into Ilastik machine learning software (Berg et al. 2019) for cell counting. The software was trained on 2–3 representative images for each region by classifying correct ‘signal’ compared to ‘background’, and then defining which objects selected were indeed correct or not. This workflow was then used for batch processing of all images for each region. The cell counts were summed for each region, per mouse, and divided by the total volume, to get cell number/mm^3^. The total volume was calculated by multiplying the depth of the z-stacks by the image dimensions (0.636 × 0.636 mm for 1024 × 1024 resolution image) for each image and then summing the volumes of each image. For the corpus callosum images, only the corpus callosum itself was selected and analyzed, and thus the volume calculations were adjusted using the size of the selected area of the corpus callosum. Representative images from both Iba1 and GFAP stained slices were manually counted by a research associate blinded to all experimental conditions and then compared to the Ilastik counts generated by the machine learning software. Strong correlations between the manual count and machine learning software were identified, *R* = 0.7764 for Iba1 slices and *R* = 0.9284 for GFAP (see Supplementary Figure S1).

### Statistical Analyses and Latent Growth Curve Modeling

SPSS 25 for Mac was used for all neuropathological results from the immunohistochemical analysis and for two of the behavioral measures (FS and DT). Two-way ANOVAs with injury (RmTBI; sham) and sex (male; female) as factors were run, and the results were considered significant if *P* < 0.050. For all displayed graphs, means are presented by the bars ± the standard error (SE) bars. For the chronic repeated behavioral analyses, we used Mplus 7.4 for Mac (Muthén and Muthén 2012) to understand how time to cross the beam, distance traveled in the open field, time spent in open arms of the EPM, percent time with the novel object, and telomere length changed over time. Specifically, we sought to determine the individual differences in the trajectories of time to cross, distance traveled, time spent in open arms, percent time with the novel object, and telomere length changed with injury (sham or RmTBI) over time. To start, we estimate a univariate latent growth curve model (LGCM) to assess the complexity of change in our variables (Preacher et al. 2008) using LGCMs to examine potential differences in the growth trajectories of our variables as a factor of injury (sham or RmTBI). To assess the level of complexity in our variables, we start by specifying an LGCM with an intercept only, representing static differences between subjects (mean levels). Next, we added a slope (degree of change throughout the course of the experiment), followed by the inclusion of a quadratic term (two levels of change throughout the course of the experiment), representing the dynamic nature of the variables. For a similar approach, see (Griep and Vantilborgh 2018). This type of LGCM was used to determine whether there is statistically significant variance in the growth parameters of a variable so that variance in these growth factors can potentially be accounted for by the application of sham versus RmTBI. To decide which model (intercept only, intercept and slope, or intercept, slope, and quadratic term) fitted the data best, we compared the competing models using the sample-size-adjusted Bayesian information criteria (ABIC). Lower levels of the ABIC suggest better fitting models (Aiken et al. 1991). After having identified best fitting univariate LGCM for each variable, we saved the growth parameters of the best fitting univariate LGCM for each variable for shams and RmTBIs using the SAVEDATA and SAVE = FSCORES command in Mplus 7.4 (Muthén and Muthén 2012). By doing so, we could compare these growth parameters using MANCOVA with injury (sham or RmTBI) as factor and sex (male or female) as control variables. The data used in this analysis can be found on DOI: 10.17605/OSF.IO/PQMKR.

## Results

### Chronic Behavioral and Telomere Length Analysis

To assess the level of complexity of the LGCM for our variables, we started by specifying separate univariate LGCMs for each focal variable with only an intercept (i.e., no change over time) as latent growth factors. Next, we included a linear term to the model (i.e., linear change over time), followed by a quadratic term (i.e., curvilinear change over time). We compared competing model using the sample-size-adjusted Bayesian information criteria (ABIC), with lower ABIC values indicating better model fit. As can be seen from Table 1, we found that time spent in open arms changed linearly over time, whereas percent time with the novel object, telomere length, time to cross the beam, and distance traveled changed in a curvilinear manner over time.

**Table 1:** Model comparison of separate univariate LGCMs

Model	ABIC
Time spent in the open arms	
Intercept only	985.46
**Intercept and slope**	**976.73**
Intercept, slope, and quadratic term	995.69
Percent time with the novel object	
Intercept only	595.77
Intercept and slope	592.32
**Intercept, slope, and quadratic term**	**569.88**
Telomere length	
Intercept only	1000.89
Intercept and slope	943.47
**Intercept, slope, and quadratic term**	**941.14**
Time to cross the beam	
Intercept only	637.15
Intercept and slope	621.22
**Intercept, slope, and quadratic term**	**619.31**
Distance traveled	
Intercept only	1274.03
Intercept and slope	1245.39
**Intercept, slope, and quadratic term**	**1244.62**

Note: bolded model represents the selected best fitting level of complexity for the LGCM.

**Figure 1:**
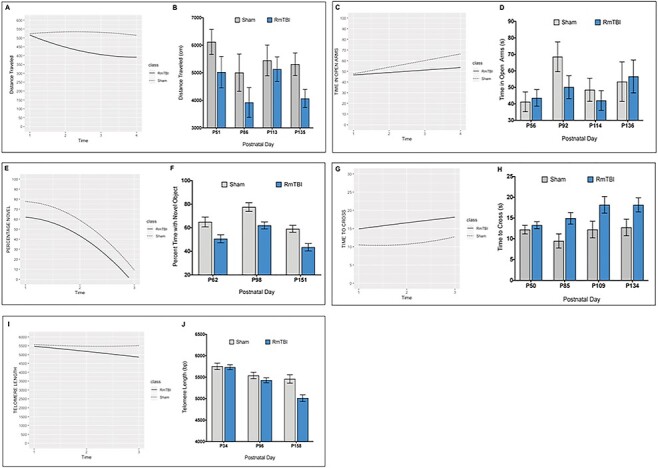
(*A*) Displays a plot of the calculated intercept, slope, and quadratic term for distance traveled in the open field. (*B*) Bar graphs displaying distance traveled in the open field at each time point. (*C*) Displays a plot of the calculated intercept and slope term for time spent in the open arms of the EPM. (*D*) Bar graphs displaying time spent in the open arms of the EPM at each time point. (*E*) Displays a plot of the calculated intercept, slope, and quadratic term for percentage time spent with the novel object in the NCM task. (*F*) Bar graphs displaying percent time with the novel object at each time point. (*G*) Displays a plot of the calculated intercept, slope, and quadratic term for time to cross the tapered beam. (*H*) Bar graphs displaying time to cross the beam at each time point. (*I*) Displays a plot of the calculated intercept, slope, and quadratic term for telomere length. (*J*) Bar graphs displaying the average telomere length at each time point. Note that negative slopes denote a decrease at that time point, positive slopes denote an increase at that time point, and slopes of zero indicate no change at that time point. Negative quadratic terms denote a further decrease when following a negative slope, whereas negative quadratic terms denote a negative inflection point when following a positive slope. Positive quadratic terms denote a further increase when following a positive slope, whereas positive quadratic terms denote a positive inflection point when following a negative slope.

First for the sham and RmTBI distance traveled (see Fig. 1*A*), we found significant differences in the intercepts (*P* < 0.001, adjusted *R^2^* = 0.721), slopes (*P* < 0.001, adjusted *R*^2^ = 0.554), and quadratic terms (*P* < 0.001, adjusted *R*^2^ = 0.627) between shams and RmTBIs; distance traveled was shorter at baseline for RmTBIs compared to shams and continued to shorten over time for RmTBIs compared to shams. Second, for the sham and RmTBI time spent in open arms (see Fig. 1*C*), we found no significant differences in the intercepts (*P* = 0.889, adjusted *R*^2^ = 0.087) and slopes between shams and RmTBIs (*P* = 0.559, adjusted *R*^2^ = 0.074); both groups spent an equal amount of time in the open arms at baseline and increased over time. Third, for the sham and RmTBI percent time with novel object (see Fig. 1*E*), we found significant differences in the intercepts between shams and RmTBIs (*P* < 0.001, adjusted *R*^2^ = 0.644); percent time with novel object was higher at baseline for shams compared to RmTBIs. However, we found no significant differences in slopes (*P* = 0.748, adjusted *R*^2^ = 0.098) and quadratic terms (*P* = 0.807, adjusted *R*^2^ = 0.084) between shams and RmTBIs; both groups decrease exponentially in percent of time with novel object over time. Fourth, for the sham and RmTBI time to cross the beam (see Fig. 1*G*), we found no significant differences in the intercepts (*P* = 0.636, adjusted *R*^2^ = 0.027), slopes (*P* = 0.196, adjusted *R*^2^ = 0.061), and quadratic terms (*P* = 0.315, adjusted *R*^2^ = 0.020) between shams and RmTBIs. Finally, for the sham and RmTBI telomere length (see Fig. 1*I*), we found no significant differences in the intercepts (*P* = 0.665, adjusted *R*^2^ = 0.060) and slopes (*P* = 0.657, adjusted *R*^2^ = 0.055) between shams and RmTBIs. However, we found significant differences in quadratic terms (*P* < 0.001, adjusted *R*^2^ = 0.445) between sham and RmTBIs; telomeres shortened exponentially faster over time for RmTBIs compared to shams.

### FS and DT Measures

#### Forced Swim

**Figure 2:**
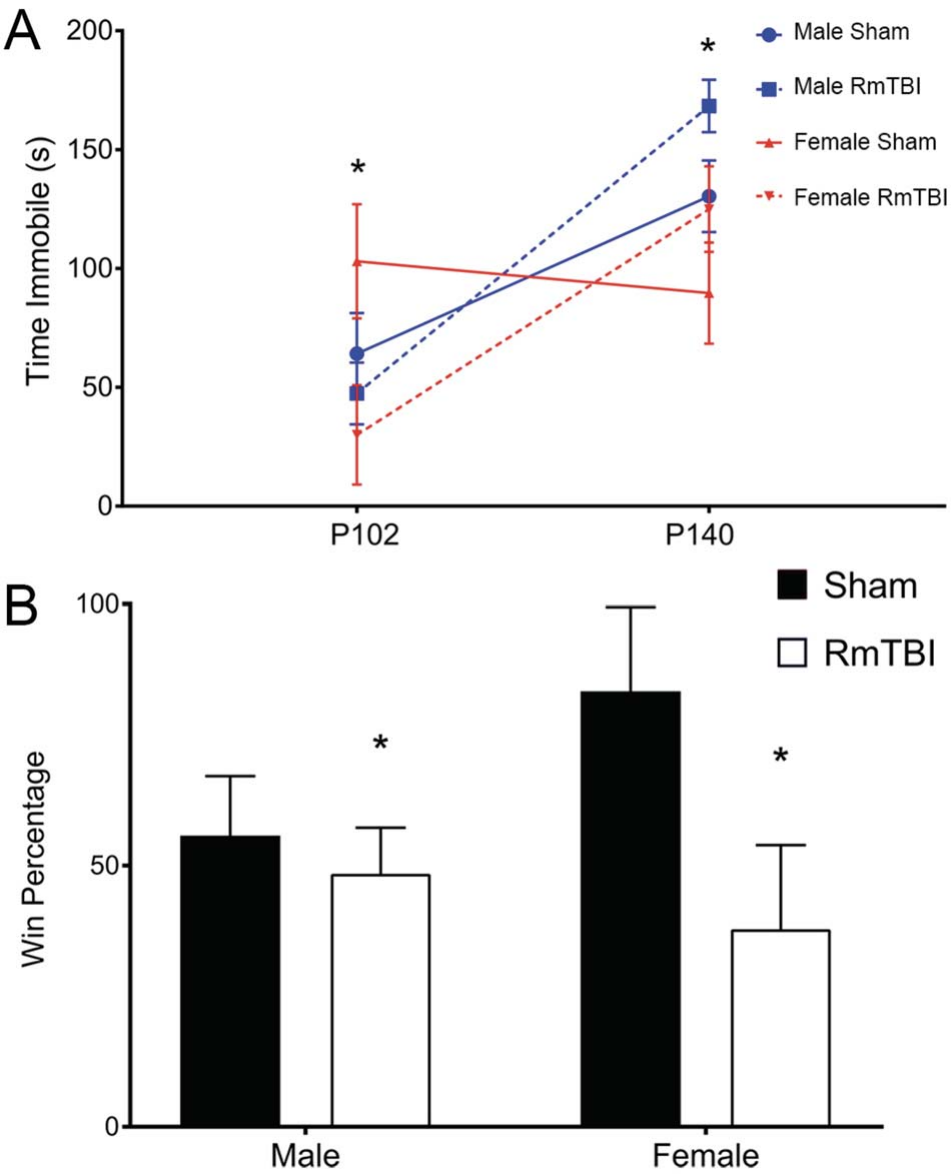
Graphs displaying the results of the (*A*) forced swim task which displays the average time spent immobile at the 2 time points (P102 and P140) and (*B*) the DT task which displays the average win percentage. Means ± SE shown (^*^) indicates a main effect of injury, *P* < 0.05.

At P102, following the 20th mTBI, results from the two-way ANOVA for time spent immobile in the FS task found that animals in the RmTBI group exhibited reductions in depressive-like behavior when compared to animals in the sham group. This was evident by the significant main effect of injury, *F*(1, 23) = 5.390, *P* = 0.032, *η*_p_^2^ = 0.221. There was no effect of sex, *F*(1, 23) = 0.309, *P* = 0.585, *η*_p_^2^ = 0.016, nor a significant interaction, *F*(1, 23) = 2.130, *P* = 0.161, *η*_p_^2^ = 0.101. Interestingly, this was reversed at P141, following the 30th mTBI where animals in the RmTBI group displayed increased depressive-like behavior as indicated by significant increases in the time spent immobile. Results from the two-way ANOVA demonstrate a main effect of injury, *F*(1, 23) = 4.655, *P* = 0.044, *η*_p_^2^ = 0.197, and also a main effect of sex, whereby males exhibited higher levels of depressive-like behavior than females, *F*(1, 23) = 6.128, *P* = 0.023, *η*_p_^2^ = 0.244. The interaction was not significant, *F*(1, 23) = 0.006, *P* = 0.939, *η*_p_^2^ = 0.000 (see Fig. 2*A*).

#### Dominance Tube

Results from the two-way ANOVA for win percentage in the DT found a main effect of injury, *F*(1, 23) = 4.173, *P* = 0.050, *η*_p_^2^ = 0.173, demonstrating that animals in the RmTBI group were actually more timid and less aggressive than animals in the sham group. There was no effect of sex, *F*(1, 23) = 0.367, *P* = 0.551, *η*_p_^2^ = 0.018, nor a significant interaction, *F*(1, 23) = 2.622, *P* = 0.120, *η*_p_^2^ = 0.116 (see Fig. 2*B*).

#### Neuropathological Results

**Figure 3:**
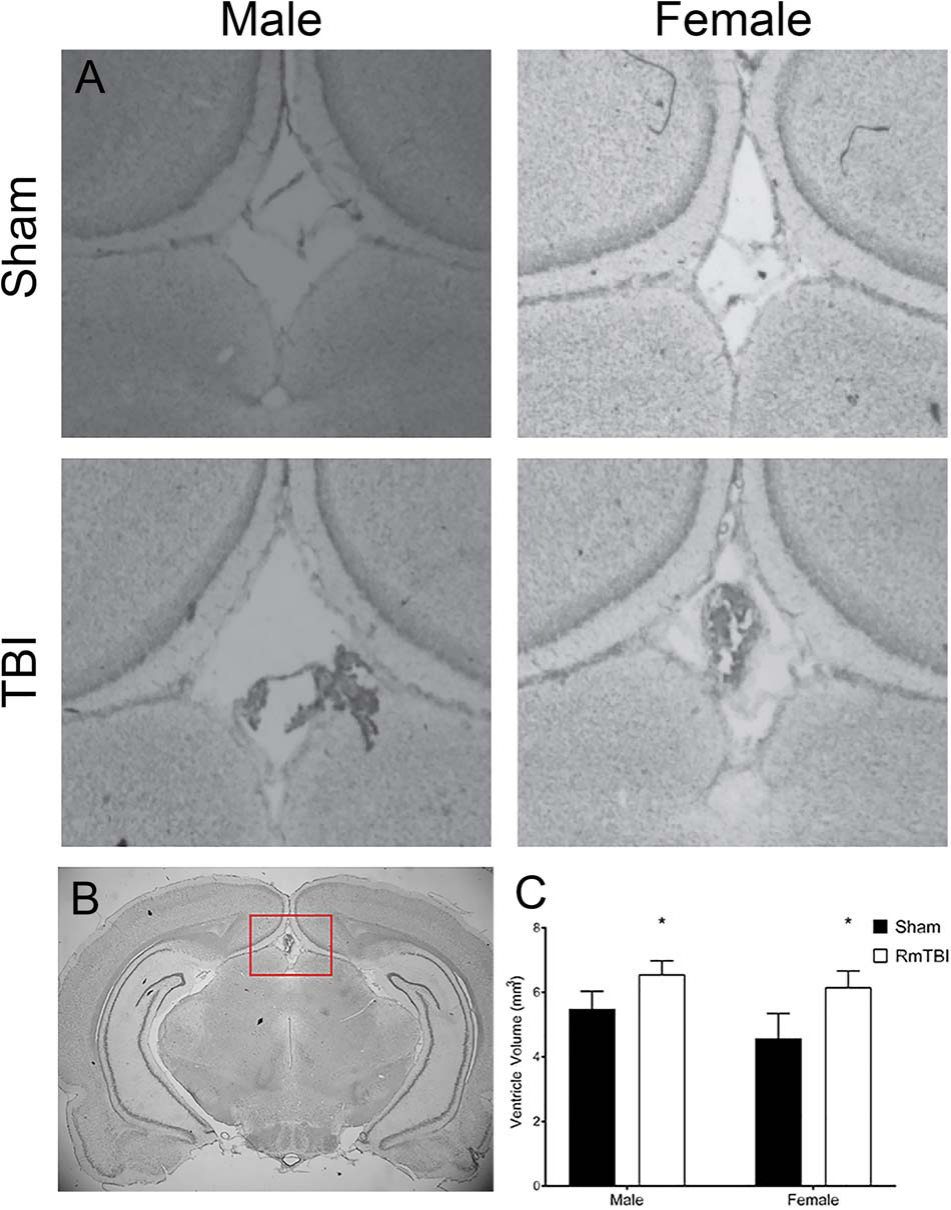
Quantitative analysis of RmTBI-induced changes in ventricle size of male and female rats. (*A*) Micrographs of cresyl violet stained ventricles from male and female rats following the RmTBI protocol. (*B*) Representative brain slice indicating the region of analysis. (*C*) Quantification of ventricle size; bars represent means ± SEM, and ^*^ indicates *P* < 0.05.

**Figure 4:**
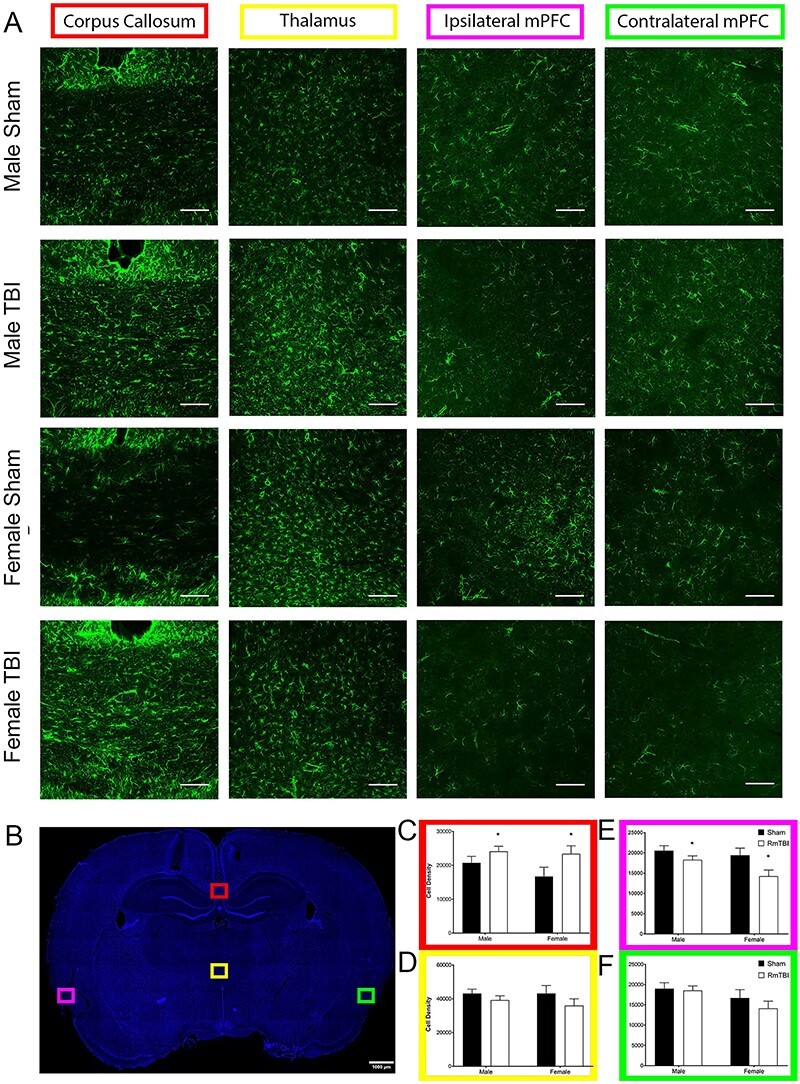
Quantitative analysis of RmTBI-induced changes in astrocytes in male and female rats. (*A*) Immunofluorescence micrographs of GFAP labeled astrocytes in the thalamus, corpus callosum, and ipsilateral/contralateral medial prefrontal cortex from male and female rats following RmTBI protocol. Each line represents 100 μm. (*B*) Representative brain slice indicating regions of analysis in *A*. (*C*–*F*) Quantification of GFAP+ cell density in the corpus callosum (*C*), thalamus (*D*), ipsilateral medial prefrontal cortex (*E*), and contralateral medial prefrontal cortex (*F*); bars represent means ± SEM, and ^*^ indicates *P* < 0.05.

**Figure 5:**
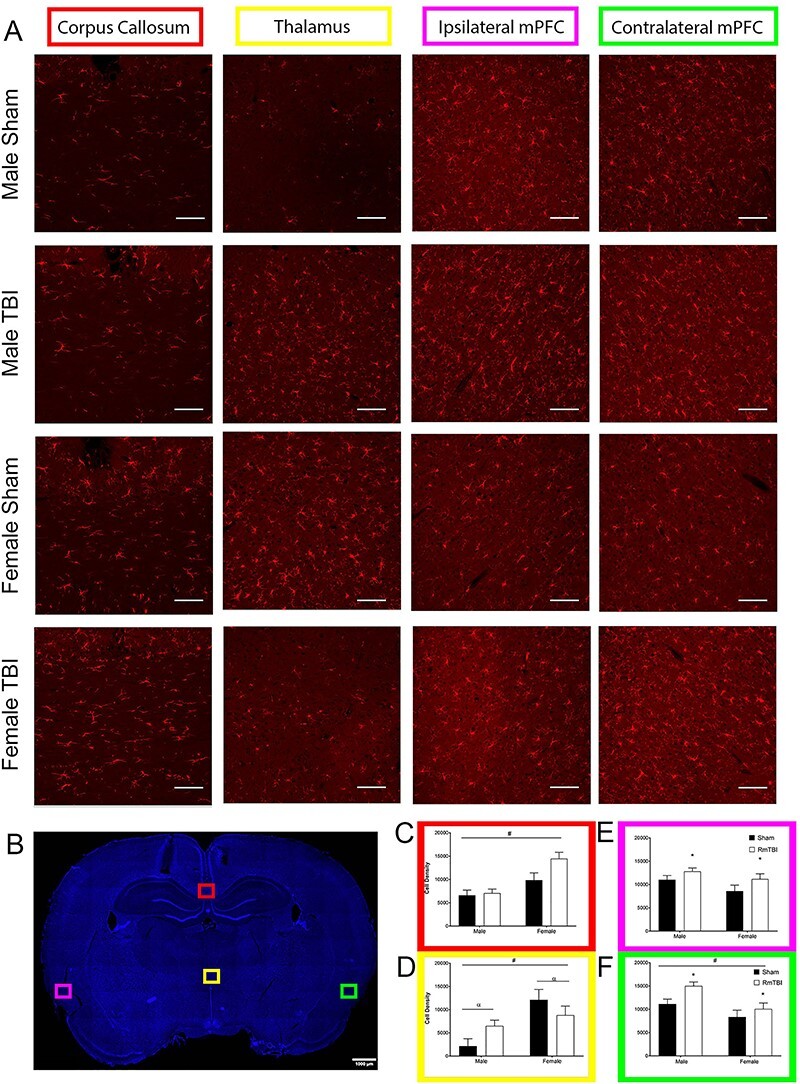
Quantitative analysis of RmTBI-induced microglial changes in male and female rats. (*A*) Immunofluorescence micrographs of IBA1 labeled microglia in the thalamus, corpus callosum, and ipsilateral/contralateral medial prefrontal cortex from male and female rats following RmTBI protocol. Each line represents 100 μm. (*B*) Representative brain slice indicating regions of analysis in *A*. (*C*–*F*) Quantification of IBA1+ cell density in the corpus callosum (*C*), thalamus (*D*), ipsilateral medial prefrontal cortex (*E*), and contralateral medial prefrontal cortex (*F*); bars represent means ± SEM, and ^*^ indicates *P* < 0.05.

Graphical representation of the neuropathological results, along with representative images of staining quality within each region, can be found in Figures 3–5.

#### Ventricle Size

Results from the two-way ANOVA for ventricle size demonstrated that RmTBI animals had larger ventricles than sham animals (main effect of injury, *F*(1, 22) = 4.955, *P* = 0.038, *η*_p_^2^ = 0.199), but there was no main effect of sex, *F*(1, 22) = 1.201, *P* = 0.286, *η*_p_^2^ = 0.057, nor a significant interaction, *F*(1, 22) = 0.188, *P* = 0.669, *η*_p_^2^ = 0.009 (see Fig. 3).

#### GFAP

In the corpus callosum, the two-way ANOVA demonstrated a main effect of injury, *F*(1, 22) = 5.412, *P* = 0.031, *η*_p_^2^ = 0.222, with RmTBI animals exhibiting elevated levels of GFAP, but we failed to identify a significant effect of sex, *F*(1, 22) = 1.175, *P* = 0.292, *η*_p_^2^ = 0.058, or a significant interaction, *F*(1, 22) = 0.576, *P* = 0.547, *η*_p_^2^ = 0.029. Within the thalamus, there were no main effects or significant interactions, *P*’s > 0.050. For the ipsilateral mPFC, we identified a main effect of injury, *F*(1, 22) = 7.058, *P* = 0.016, *η*_p_^2^ = 0.271, whereby RmTBI animals actually exhibited reductions in GFAP density when compared to sham animals. Again, we failed to identify a main effect of sex, or a significant interaction, *P*’s > 0.050. Finally, there were no significant differences in the contralateral mPFC for GFAP density; however, there was a trend towards a significant main effect of sex, *F*(1, 22) = 4.159, *P* = 0.056, *η*_p_^2^ = 0.180, with male rats exhibiting higher GFAP densities than female rats (see Fig. 4).

#### IBA1

In the corpus callosum, the two-way ANOVA identified a main effect of sex, *F*(1, 22) = 18.192, *P* < 0.001, *η*_p_^2^ = 0.489, with females exhibiting higher IBA1 densities than males, and a trend towards a main effect of injury, *F*(1, 22) = 4.138, *P* = 0.056, *η*_p_^2^ = 0.179, with RmTBI rats also exhibiting elevated IBA1 densities. The interaction was not significant, *F*(1, 22) = 2.755, *P* = 0.113, *η*_p_^2^ = 0.127. Interestingly in the thalamus, the two-way ANOVA also demonstrated a main effect of sex, *F*(1, 22) = 11.717, *P* = 0.003, *η*_p_^2^ = 0.381, with females again exhibiting higher IBA1 densities than males. However, in the thalamus, there was also a significant sex by injury interaction, *F*(1, 22) = 4.546, *P* = 0.046, *η*_p_^2^ = 0.193, whereby IBA1 was elevated in male rats with RmTBI, but reduced in female rats with RmTBI. In the ipsilateral mPFC, RmTBI increased IBA1 density as demonstrated by the significant main effect of injury, *F*(1, 22) = 4.595, *P* = 0.045, *η*_p_^2^ = 0.195. There was not a main effect of sex, nor a significant interaction, *P*’s > 0.050. Finally, in the contralateral mPFC, there was a main effect of sex, *F*(1, 22) = 10.792, *P* = 0.004, *η*_p_^2^ = 0.375, with males exhibiting higher IBA1 densities than females, and a main effect of injury, *F*(1, 22) = 5.679, *P* = 0.028, *η*_p_^2^ = 0.240, with RmTBI also increasing IBA1 density. There were no significant interactions in the contralateral mPFC (see Fig. 5).

## Discussion

The purpose of this study was to examine the progressive changes in behavior, and the neuropathological outcomes, associated with chronic RmTBI through adolescence and early adulthood in male and female Sprague Dawley rats. Most animal studies examining RmTBI use 2–7 mTBIs spaced over days or weeks (e.g. Ojo et al. 2013; McAteer et al. 2016; Mychasiuk et al. 2016a, 2016b; Gao et al. 2017; Salberg et al. 2018; Yamakawa et al. 2019). This innovative study examined 30 mTBIs over 15 weeks (~4 months), covering all of adolescence and early adulthood. Although epidemiological evidence suggests that chronic exposure to concussive and sub-concussive impacts may have negative effects on neurological function, very few preclinical studies have administered continuous mTBIs, and even less so, across such an important developmental period. Although we found evidence for neurological impairment, the outcomes were not as substantial as one would have predicted in response to 30 mTBIs, nor did they align with complete behavioral patterns that have been associated with CTE.

### Behavioral Assessment

Motor behavior was examined at 4 time points using the tapered beam walking task and the open field. Although rats in the RmTBI group exhibited motor impairment, as indicated by longer times to cross the beam at all 4 time points, this motor deficit did not worsen with progressive injuries. Rather, motor deficits appeared to plateau following the 22nd mTBI and remain stable from 3 on. This finding of minor, nonprogressing motor impairment is corroborated by our results from the open field task. Although animals in the RmTBI group were less active than animals in the sham group, this impairment did not worsen over time or with progressive injuries. Similarly, short-term working memory and cognitive function, as measured with the NCM task, were significantly impaired in RmTBI animals at all three time points examined. Of particular importance is the finding at P151, which occurred 18 days after the final mTBI, a period where one would have predicted functional recovery (Mccrory et al. 2018). These findings suggest that the repetitive injuries did induce persistent but stable deficits in motor behavior and short-term working memory.

Interestingly, and contrary to expectations based upon clinical report (Montenigro et al. 2014), rats exposed to RmTBI did not exhibit changes in anxiety-like behavior (at any measurement point) and were actually less aggressive and more timid than their sham counterparts. Similar to ours, studies have consistently reported no changes in anxiety-like behavior following RmTBI, both at the acute and chronic time points (Fidan et al. 2016; McAteer et al. 2016). To our knowledge, this is the first preclinical study to examine social status and aggressive-like behavior within the DT following chronic RmTBI. The findings of reduced social status and increased withdrawal/retreat behavior in the DT test contradict many of the CTE-related claims that a hallmark trait of repetitive head injuries is increased aggression and emotionally “explosive” behavior (Montenigro et al. 2014). Finally, RmTBI-induced changes in depressive-like behavior varied according to the time point analyzed. When examined in late-adolescence/early adulthood (P102; following the 20th mTBI), rats in the RmTBI group exhibited reductions in depressive-like behavior as indicated by reduced time immobile. However, when re-evaluated in adulthood (P140; 7 days following the last mTBI), injured animals displayed increased levels of depressive-like behavior. In clinical populations it is not uncommon to see age-dependent variations in depressive symptomology; aged individuals are more resilient to mTBI-induced depression when compared to younger adults (Rapoport et al. 2003), and older teens (16–17) are more susceptible than younger teens (12–14) (Chrisman and Richardson 2013). This exemplifies the heterogeneous nature of mTBI and importance of continuous monitoring of symptomologies across the life span.

Consistent with many of our previous studies (Hehar and Mychasiuk 2016; Hehar et al. 2017; Wright et al. 2018), RmTBI significantly reduced TL, with LGC analysis demonstrating that telomeres shortened exponentially faster over time for RmTBIs compared to shams. This finding is in accordance with theories that chronic mild TBI exposure reduces long-term neuroplasticity and accelerates aging. Smith et al. have postulated that each successive mTBI leads to acute neuropathology that may or may not completely resolve, and this accumulating pathology leads to earlier onset cognitive impairment and accelerates the rate at which the brain typically ages (Smith et al. 2013). Our results provide corroborating evidence for this theory, given that telomere shortening has been used as a biological marker for cellular aging (Allsopp et al. 1992; Klapper et al. 2001; Blasco 2005).

### Neuropathological Assessment

In line with some of the clinical reports regarding CTE (McKee et al. 2016), we did identify enlargement of the lateral and third ventricles in RmTBI animals. In the acute post-TBI periods, ventricle size is generally reduced in response to tissue expansion associated with cerebral edema and increased parenchymal volume (Toth et al. 2012; Bigler 2013). However, this is usually transient in mTBI and quickly subsides. While enlarged ventricles are more commonly identified in the chronic phases following moderate or severe TBI (Poca et al. 2005), they have also been associated with aging and neurological diseases (Jackson et al. 2011; Apostolova et al. 2012) and have been described in very young animals (<P21) exposed to RmTBIs (Huh et al. 2007). Although the mechanisms driving ventricle enlargement often vary, in RmTBI it is hypothesized that repeated increases in intracranial pressure result in ventricle expansion (Mawdsley and Ferguson 1963; Smith et al. 2013). Nevertheless, the literature regarding the prolonged changes to ventricle size in preclinical models is sparse and requires further investigation to fully understand the mechanisms and the significance of these changes.

GFAP is a brain-specific filament protein that is normally expressed by astrocytes and plays important roles in physiological astrocyte biology. GFAP expression can increase in response to TBI as it aids in glial scar formation (Di Giovanni et al. 2005). The increase in GFAP+ cells in the CC is consistent with preclinical studies that demonstrate more substantial pathological deficits in white matter as opposed to deeper structures like the hippocampus (McAteer et al. 2016), and clinical studies illustrating that white matter is much more susceptible to concussion-based injuries than gray matter (for review see Narayanan 2017). The significant reduction in GFAP in the ipsilateral cortex of RmTBI animals is a more perplexing finding, as the vast majority of TBI studies report increases in GFAP levels following injury (e.g. Ojo et al. 2013; Mannix et al. 2014; Xu et al. 2016). Interestingly, and in line with our findings of chronic repetitive injury, a recent in vitro study found that astrocytes developed mechano-sensitivity to repeated impact conditions and actually demonstrated reductions in mRNA for genes such as GFAP, collagen, and versican (Walker et al. 2019). It is therefore possible that the repetitive injuries also sensitized the astrocytic response. Finally, given that GFAP is considered an acute serum/CSF biomarker as it is believed to peak in the hours to days post-mTBI, it is not surprising that we failed to identify changes in GFAP expression for RmTBI animals in the thalamus and the contralateral mPFC, as this analysis occurred 25 days following the last RmTBI. Moreover, chronic activation of GFAP would have been expected in response to glial scar formation and astrogliosis (Burda et al. 2016), but mTBI is more commonly associated with diffuse axonal injury and not necessarily scar-producing focal lesions.

As the majority of chronic RmTBI studies to date have focused solely on male rodents (Mannix et al. 2014; Petraglia et al. 2014; McAteer et al. 2016; Xu et al. 2016; Gao et al. 2017), the significant sex differences in IBA1 expression in the CC, thalamus, and mPFC, following RmTBI, highlight the importance of studying both males and females. Despite having similar RmTBI-induced behavioral manifestations, it is clear that the neuropathological outcomes are very different between sexes. While it is well accepted that microglia play key roles in the neuroinflammatory response, rapidly proliferating and responding to neurological insult such as TBI, it has only recently been shown that microglia exhibit robust sex differences in number, morphology, and maturation (Schwarz et al. 2012; Hanamsagar et al. 2017; Villa et al. 2018) and has been implicated in sex-specific behavior (Bordt et al. 2019). Given this, and the significant sex differences in brain maturation that occur throughout adolescence and early adulthood (De Bellis et al. 2001), it should not be surprising that these important cells exhibit sexually dimorphic responses to RmTBI during this period.

The thalamus is a highly connected brain structure, with significantly more corticothalamic projections than thalamocortical projections. Therefore, post-RmTBI thalamic microglia activation is likely due to anterograde transneuronal and axonal damage and neurodegeneration (Donat et al. 2017). If this is the case, the stark sex differences in thalamic IBA1 expression may be associated with the neuroprotective capacity of estrogen to attenuate the diffuse neuronal and axonal damage associated with these mTBIs (Raghava et al. 2017). Interestingly, although levels of IBA1+ cells were similar for shams across both the ipsilateral and contralateral mPFC cortices, the RmTBI-induced increases in IBA1+ cells were substantially greater in the contralateral cortex than the ipsilateral. Given that all 30 mTBIs were administered to the ipsilateral cortex, this finding highlights the diffuse nature of the injury and suggests that deficits spread throughout the cortex, and this may account for some of the persistent functional impairments.

## Conclusions

This study provides further evidence that additional factors above and beyond trauma and repetitive head injuries are required to trigger CTE and the associated neurochemical cascades. While RmTBI did induce some neurological deficits and neuropathological changes, the outcomes were not as substantial as one would have predicted in response to 30 mTBIs and highlight the exceptional plasticity of the brain (Kolb and Whishaw 1998; Kolb and Gibb 2011). It is also important to note that within this study, the mTBIs were administered on a regimented schedule, which purposely did not allow the brain adequate time between injuries to fully recover. If appropriate neurological rest were to be allowed, it is possible that the detrimental effects we identified could have been minimized. In addition, the number of female rats utilized was notably smaller than the number of male rats and may have limited our ability to ascertain sex differences in the behavioral outcomes. The study is limited by a lack of Tau or phospho-Tau staining as this would have added to the understanding of RmTBI pathology within the oscillating CTE landscape. However, given the vast majority of RmTBI studies in rodents have failed to identify changes in Tau pathology unless using a tau-based transgenic animal (Ojo et al. 2013, 2016; McAteer et al. 2016; Gao et al. 2017), this may not have provided additional benefit. In addition, neuropathological assessments across the continuum would have also increased our understanding of how RmTBI neuropathology progresses with increasing injury burden and within the context of brain maturation. Given that RmTBI has been implicated as a risk factor for a plethora of neurological disorders, and fear is growing regarding participation in collision/contact sports, especially for children and adolescents, additional studies such as this are needed to further understand the persistent consequences of RmTBI.

## Notes

*Conflict of Interest*: The authors have no competing financial interests with respect to the work described here.

## Funding

The authors would like to thank the Alberta Children’s Hospital Research Institute, Canadian Institute of Health Research, and Natural Sciences and Engineering Research Council for their financial contributions. *Conflict of Interest*: The authors have no competing financial interests with respect to the work described here.

## Supplementary Material

Supplementary_Figure_1_tgaa002

Supplementary_File_tgaa002
