# Supplementary figures and images for: Examining the Progressive Behavior and Neuropathological Outcomes Associated with Chronic Repetitive Mild Traumatic Brain Injury in Rats
Source: Cereb Cortex Commun. 2020 Feb 20;1(1):tgaa002. doi: 10.1093/texcom/tgaa002 (PMC8152839; doi:10.1093/texcom/tgaa002)

Iba1

GFAP

Manual

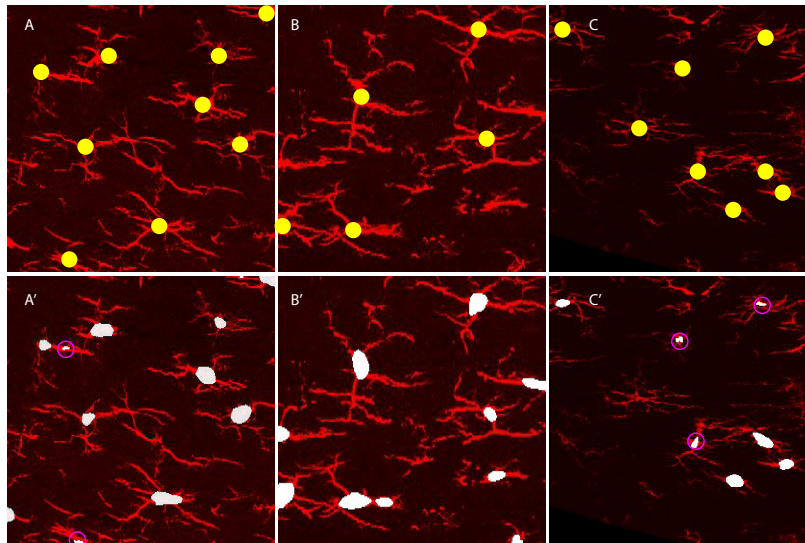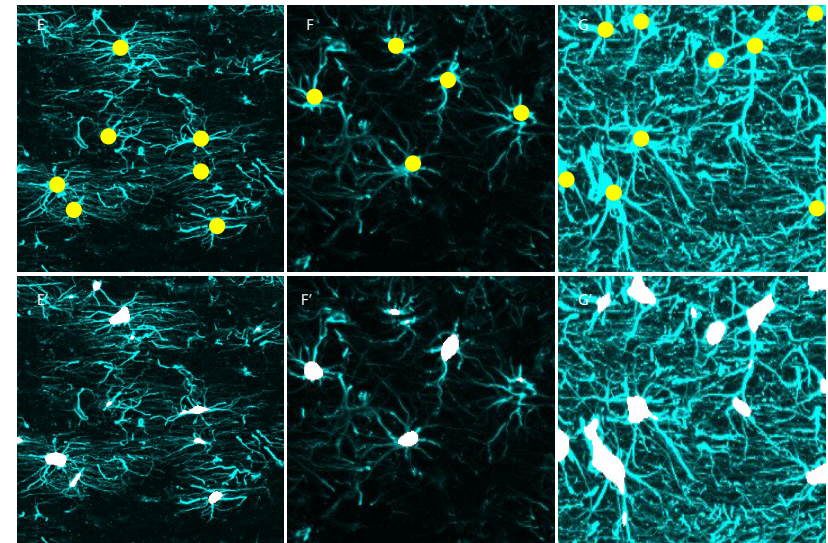

Ilastik

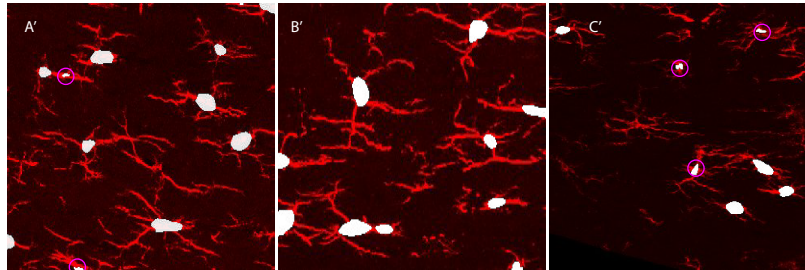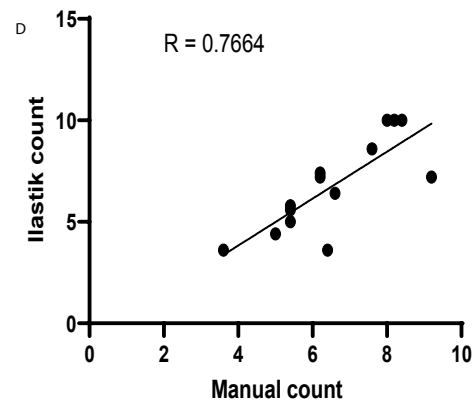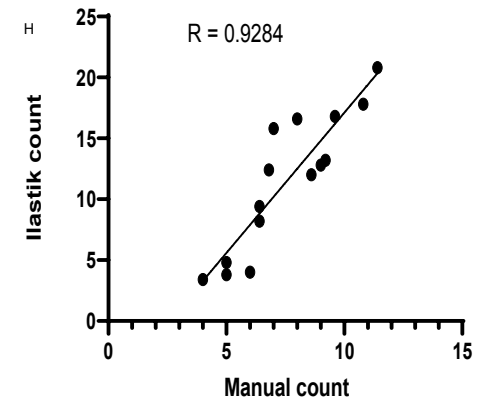

Supplement: Supplementary_Figure_1_tgaa002 [file supplementary_figure_1_tgaa002.pdf]
