# Supplementary material for: Examining the Progressive Behavior and Neuropathological Outcomes Associated with Chronic Repetitive Mild Traumatic Brain Injury in Rats
Source: Cereb Cortex Commun. 2020 Feb 20;1(1):tgaa002. doi: 10.1093/texcom/tgaa002 (PMC8152839; doi:10.1093/texcom/tgaa002)
Supplement: Supplementary_File_tgaa002 [file supplementary_file_tgaa002.docx]

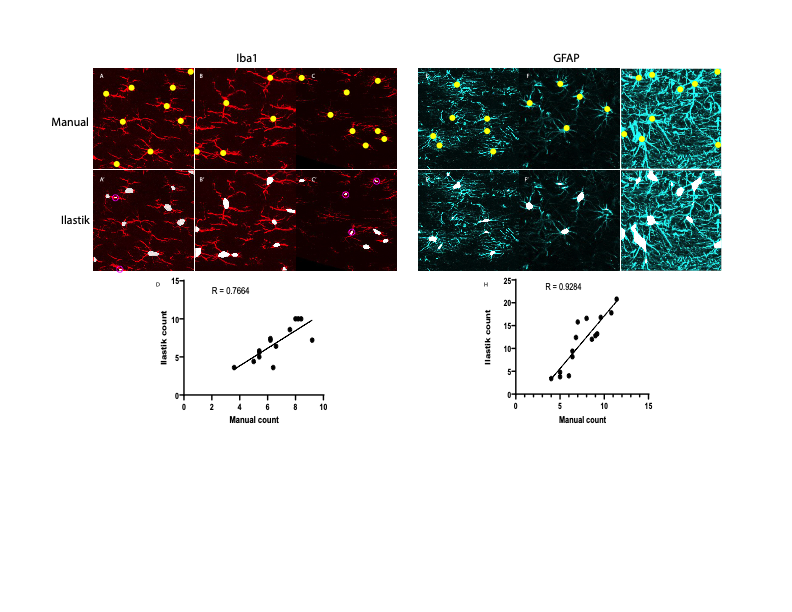


**Supplementary Figure 1**. Illustrative representation of cell counts and correlations generated manually and via the Ilastik machine learning software for Iba1 and GFAP labelled cells.
